# ESR1 mutations in breast cancer

**DOI:** 10.18632/aging.101165

**Published:** 2017-01-25

**Authors:** Florian Clatot, Laetitia Augusto, Frédéric Di Fiore

**Affiliations:** Inserm U1245, Rouen, France

**Keywords:** ESR1 mutations, breast cancer, circulating DNA

In the area of personalized medicine, the main challenge of treating metastatic breast cancer (BC) remains to improve overall survival without deteriorating quality of life. Around 70% of breast tumors express estrogen receptors (ER+), which makes them usually sensitive to a hormonal blockage. Hormonal therapy is commonly the recommended first-line treatment for ER+ metastatic BC since it can provide a tumor growth arrest with few side-effects [1]. Aromatase inhibitors (AIs) is the actual backbone of hormonal treatment in the metastatic setting for many patients. The rationale of AIs is the inhibition of estrogene synthesis in peripheral tissues leading to complete lack of activation of the ER. If numerous mechanisms of AI resistance may occur, the key role of the estrogen receptor gene (*ESR1*) mutations was recently investigated. Several hot-spot mutations have been reported, usually modifying the ligand biding domain of the ER, leading to a ligand-independent receptor activity. Collectively, the 5 main mutations (D538G, E380Q and D537S/N/C) represent more than 80% of the *ESR1* mutations. These mutations are an acquired molecular event since they are almost absent in primary BC tumour (<2%) but occur in metastatic tissues in around 25-30% of cases secondary to AI exposure [2]. At a glance, the emergence of *ESR1* mutations is a marker of AI resistance.

Instead repeated biopsies of metastases, it has been demonstrated that the detection of *ESR1* mutations in circulating tumor DNA (ctDNA) by digital droplet PCR (ddPCR) is sensitive and highly correlates to the *ESR1* mutational status in tumor tissue [3]. Thus, plasma samples from ER+ metastatic BC patients can be regarded as liquid biopsies and might help choosing the right treatment to the right patient at the right time in the near future. Indeed, a growing amount of evidence has supported the potential clinical utility of *ESR1* mutational status assessment. We have recently shown that *ESR1* mutation can raise during first-line AI treatment in the metastatic setting, with 75% of the *ESR1* mutated patients having a detectable circulating mutation at least 3 months before clinical progression [4]. Several studies have reported that the detection of circulating *ESR1* mutations was an independent factor of poor prognosis both in progression-free survival [5,6] and in overall survival [4,6]. In terms of treatment, if the use of CDK or mTOR inhibitors in association with hormonal therapy provides a significant improvement for ER+ metastatic BC, their benefit in case of *ESR1* mutation is not yet established. Prospective studies are needed to determine the best therapeutic options when circulating *ESR1* mutations occurre during AI exposure. The next act of the “ESR1 saga” will be played at a molecular level. Indeed, it has been reported that the resistance to hormonal treatment is different when considering each individual *ESR1* mutation [7]. Toy et al. performed a functional analysis using MCF7 cells and confirmed that the ER auto-activation associated with *ESR1* mutations was maximal in Y537S mutants. Furthermore, the estrogen-independent activity of mutant receptors was higher in frequent mutants – in particular Y537S – than in less frequent mutants such as E380Q. Interestingly, they also showed that fulvestrant, an ER inhibitor currently used in clinical practice, may reverse resistance induced by *ESR1* mutations [5] with a differential effect according to mutations; the Y537S mutant requiring the highest dose of fulvestrant to achieve growth inhibition of MCF7 cells compared to other mutants. These results were confirmed in MCF7 derived xenografts exposed to fulvestrant, for which fulvestrant provided a growth arrest in all mutants tested (wild type, E380Q, S463P, Y537C/N or D538G) but not for the Y537S one. In contrast, the AZD9496 compound - an ER inhibitor with available phase I data - inhibited successfully D538G and Y537S mutated MCF7-derived xenografts. Interestingly, these data are in line with a retrospective analysis of a large clinical trial, in which patients harboring the D538G mutation benefited from the addition of everolimus (mTOR inhibitor) to exemestane (aromatase inhibitor) for metastatic breast cancer patients progressing on AI, while it was not the case for patients with Y537S mutations [6].

In conclusion, *ESR1* mutations have recently emerged as a key mechanism of AI resistance in ER+ metastatic BC. *ESR1* mutations are a new prognostic factor of poor survival which appearance can be monitored in blood sample, and for which specific drugs are on development. The recent publication by Toy et al. also underlines the need for a better understanding of the clinical outcome depending on the peculiar *ESR1* mutation observed.
